# Laboratory Evaluation of *Isaria fumosorosea* CCM 8367 and *Steinernema feltiae* Ustinov against Immature Stages of the Colorado Potato Beetle

**DOI:** 10.1371/journal.pone.0152399

**Published:** 2016-03-25

**Authors:** Hany M. Hussein, Oxana Skoková Habuštová, Vladimír Půža, Rostislav Zemek

**Affiliations:** 1 Department of Biochemistry and Physiology, Institute of Entomology, Biology Centre CAS, České Budějovice, Czech Republic; 2 Department Biosystematics and Ecology, Institute of Entomology, Biology Centre CAS, České Budějovice, Czech Republic; 3 Pests and Plant Protection Department, National Research Centre, Cairo, Egypt; Ecole des Mines d'Alès, FRANCE

## Abstract

The Colorado potato beetle, *Leptinotarsa decemlineata*, has developed resistance to most registered pesticides and has become one of the most difficult insect pests to control. Development of new biopesticides targeting this pest might solve the resistance problem and contribute to sustainable crop production. Laboratory experiments were conducted to assess the efficacy of *Isaria fumosorosea* (syn. *Paecilomyces fumosoroseus*) strain CCM 8367 against *L*. *decemlineata* when applied alone or combined with the entomopathogenic nematode *Steinernema feltia*e. The last-instar larvae of the Colorado potato beetle showed the highest susceptibility to *I*. *fumosorosea* followed by pre-pupae and pupae. The median lethal concentration (LC_50_) was estimated to be 1.03×10^6^ blastospores/ml. The strain CCM 8367 was more virulent, causing 92.6% mortality of larvae (LT_50_ = 5.0 days) compared to the reference strain Apopka 97, which caused 54.5% mortality (LT_50_ = 7.0 days). The combined application of the fungus with the nematodes increased the mortality up to 98.0%. The best results were obtained when *S*. *feltiae* was applied simultaneously with *I*. *fumosorosea* (LT_50_ = 2.0 days); later application negatively affected both the penetration rate and the development of the nematodes. We can conclude that the strain CCM 8367 of *I*. *fumosorosea* is a prospective biocontrol agent against immature stages of *L*. *decemlineata*. For higher efficacy, application together with an entomopathogenic nematode is recommended.

## Introduction

The Colorado potato beetle (CPB), *Leptinotarsa decemlineata* (Say) (Coleoptera: Chrysomelidae), is one of the most economically damaging insect pests of potatoes (*Solanum tuberosum* L.) in the USA and through much of Europe [1]. The first European population was established in France in 1922. By the end of the 20th century, the pest had become a problem all over Europe, in Asia Minor, Iran, Central Asia, and western China [2,3]. In warm and dry regions, the population of CPB regularly occurs in high abundances and develops two complete generations. Adults and larvae feed on leaves; a single insect can eat at least 100 cm^2^ of potato foliage in its lifetime. Without control measures, the beetle can cause severe reductions in tuber yield or quality (tuber size). Because of warm weather conditions during the 1990s, severe losses occurred in Germany and Poland. Consequently, insecticide use increased considerably. On average, 2–3 treatments per year were performed [4]. The CBP has developed resistance to most registered pesticides [5–8] and become one of the most difficult insect pests to control.

One possibility for regulating the pest is to use genetically modified (GM) potatoes expressing *Bacillus thuringiensis* delta-endotoxin that is toxic to CPB. GM potatoes have been registered and sold in the USA from 1995–2000 but were discontinued in response to consumer concerns about genetically modified crops [9].

A prospective solution to the CPB resistance problem could be to develop microbial biopesticides targeted against CPB as alternatives to broad-spectrum chemical insecticides. A substantial number of mycoinsecticides and mycoacaricides have been developed worldwide since the 1960s. Products based on *Beauveria bassiana* (Balsamo) Vuillemin (Hypocreales: Cordycipitaceae) (33.9%), *Metarhizium anisopliae* (Metsch.) Sorokin, (Hypocreales: Clavicipitaceae) (33.9%), *Isaria fumosorosea* (WIZE) Brown & Smith (Hypocreales: Cordycipitaceae) (5.8%), and *B*. *brongniartii* (Saccardo) (Hypocreales: Cordycipitaceae) (4.1%) are the most common among the 171 products available [10]. The targets comprise insects in the orders Hemiptera, Coleoptera, Lepidoptera, Thysanoptera, and Orthoptera, distributed among at least 48 families. A broader appreciation for the attributes of entomopathogens is envisioned, and synergistic combinations of microbial control agents with other technologies is expected to occur in the future [11].

*Isaria fumosorosea* was known as *Paecilomyces fumosoroseus* for more than 30 years and was recently transferred to the genus *Isaria* [12]. Genetic analysis demonstrated that there are at least three monophyletic groups of *I*. *fumosorosea* [13–15]. Because of the high level of genetic diversity along with the difficulties of exact identification, *I*. *fumosorosea* must be seen as a species complex, and its taxonomic revision is urgently needed [12]. It is commonly found in the soil [16] but has been reported on plants, in water, and less commonly, in air on every continent except Antarctica [12]. It has been isolated from over 40 species of arthropods, representing 10 orders. Some of the more commonly known susceptible organisms include weevils, ground beetles, plant beetles, aphids, whiteflies, psyllids, wasps, termites, thrips, and a wide variety of butterflies and moths [17–19]. It therefore has received significant attention as a possible biological control agent for several economically important insect pests of agricultural crops [20]. Like most entomopathogenic fungi, it infects its host by breaching the cuticle [21]. Various metabolites allow the pathogen to physically penetrate the host and inhibit its regulatory system. For *I*. *fumosorosea*, these include proteases, chitinases, chitosanase, and lipase [22]. These enzymes allow the fungus to breach the insect cuticle and disperse through the hemocoel. *Isaria fumosorosea* and other species within the genus also produce beauvericin [23], a compound that appears to paralyze host cells [21]. Susceptible insects exposed to blastospores and conidia of *I*. *fumosorosea* show declined growth and high levels of mortality [18]. Various strains of *I*. *fumosorosea* are successfully used in the biocontrol of many pest insects and mites, and several commercially produced mycopesticides based either on *I*. *fumosorosea* alone or in combination with other entomopathogenic species have been developed in America, Europe or Asia [12]. To our knowledge, no research on the control of CPB by this species of entomopathogenic fungus has been published.

Entomopathogenic nematodes (EPNs) in the families Steinernematidae and Heterorhabditidae (Rhabditida, Nematoda) are obligate pathogens of insects [24] and are associated with specific symbiotic bacteria of the genera *Xenorhabdus* and *Photorhabdus*, respectively [25]. Because of their ability to infect various insects [26], the possibility of mass production by industrial techniques [27] and the relative safety to non-target organisms [28] and the environment [29], EPNs represent an attractive agent for the inundative biological control of many insect pests [30]. EPNs have been shown to infect and kill the CPB [31,32]; however, their efficiency against the CPB in the field is limited by various factors, including depth of beetle pupation, beetle migration and insensitivity of the adult beetles to nematode infection [33].

Biocontrol can be improved by using combinations of different biocontrol agents [34–36]. EPNs and entomopathogenic fungi together performed more efficiently than when applied alone [37,38]. It is thus expected that simultaneous application of the fungus and nematodes could improve their performance against CPB.

The aim of our work was to assess the efficiency of a new strain of the entomopathogenic fungus *I*. *fumosorosea* isolated in the Czech Republic [39] and the entomopathogenic nematode *Steinernema feltia*e (Filipjev) (Rhabditida: Steinernematidae) strain Ustinov against *L*. *decemlineata* either applied alone or in combination.

## Materials and Methods

### Ethics Statement

This study did not involve endangered or protected species. *Leptinotarsa decemlineata* is a common pest occurring in most of potato fields so no specific permissions were required for its collection. The insects were collected from private land and the owner gave permission to conduct the study on this site.

### Plants

Potato plants, *Solanum tuberosum* L. (Solanaceae) cv. Désirée and Superior obtained from the bank of potato genetic resources at the Potato Research Institute Havlíčkův Brod, Czech Republic, were used for insect rearing and bioassays with CPB larvae. The data on the cultivars are accessible on the website http://europotato.org. The plants were grown in large pots (20 cm diameter, 18.5 cm height) containing universal horticultural substrate B (Rašelina Soběslav, Czech Republic) including minerals and fertilizers. The greenhouse was air conditioned, and maintained at a temperature of 23–25°C. No other fertilizers or pesticides were applied to the plants.

### Insects

Laboratory culture of CPB maintained at the Institute of Entomology, České Budějovice, was established from several hundred adult individuals collected from potato fields in the vicinity of České Budějovice (South Bohemia, Czech Republic, 49°N) in 2004. Larvae and adults were reared on potato plants in a greenhouse under controlled conditions (temperature 24±2°C, photoperiod 16L:8D). Every year, the culture of CPB beetles is supplemented by around 500 fresh adults collected in the field to prevent the genetic shift (degenerations of culture). Wax moth larvae, *Galleria mellonella* L. (Lepidoptera: Pyralidae) for nematode multiplication were reared in the dark at 30°C on artificial diet [40].

### Entomopathogenic fungi

The *I*. *fumosorosea* isolate originated from the horse chestnut leaf miner, *Cameraria ohridella* Decka and Dimic (Lepidoptera: Gracillariidae). The strain is deposited under number CCM 8367 as a patent culture in the Czech Collection of Microorganisms in Brno [39]. As a reference strain, we used an isolate cultured from the commercial product PreFeRal® WG (Biobest, Belgium; *I*. *fumosorosea* strain Apopka 97 as an active ingredient). Blastospores of both strains were obtained after 120 hours of submerged cultivation in growth media (glucose, maltose, starch and peptone) using an orbital shaker at 140 cycles per minute at 23°C. The number of blastospores in suspension was counted with a Bürker counting chamber (Brand, Wertheim, Germany) and adjusted to required concentration. The soaking agent Tween 80 (Sigma-Aldrich) was added to the suspension at a concentration of 0.02% (v/v).

### Entomopathogenic nematodes

*Steinernema feltia*e, strain Ustinov, originating from Izhevsk (Russia), was reared for more than 25 years under laboratory conditions using the last larval instar of *G*. *mellonella* as a host. The emerging infective juveniles (IJ) were harvested from White traps [41] and subsequently stored in water at 10°C for 10–21 days [27]. The viability of IJs was checked under a microscope before use in the experiments. The species identity of the nematode was confirmed by sequencing the ITS region of the rDNA (Genbank accession number: KT809344).

### Bioassays

#### General conditions

The experiments were conducted using polystyrene Petri dishes (9 cm in diameter, Gosselin SAS, France) lined with moist filter paper (KA 2, Amersil–FILPAP, Ltd., Czech Republic) over a period of two years. Each test described below was repeated twice or thrice; each replication was from different generation of CPB and tested 20–40 insect individuals. All of the Petri dishes containing inoculated/control individuals were placed in an incubator under controlled conditions (23±1˚C and 16L:8D photoperiod). The treated and control insects were monitored at 24-h intervals to record daily mortality for a period of seven days.

#### The efficacy of strain CCM 8367 against immature stages of *L*. *decemlineata*

Last-instar larvae, pre-pupae and pupae of CPB in the treated group and in the control group were individually placed into Petri dishes. Treated groups: each specimen was immersed in the suspension of blastospores of strain CCM 8367 at concentration 5×10^7^ spores/ml before it was placed in the Petri dish, and additionally, one milliliter of the same suspension was applied topically to the specimen. Control groups: all specimens were treated identically as described above by using distilled water and Tween 80 at a 0.02% concentration. For the last larval instar, fresh potato leaves were placed into each dish and replaced daily. Filter paper and a piece of cotton wool were moistened by distilled water daily to maintain optimal humidity inside the Petri dishes.

#### Dose-response of CPB larvae to *I*. *fumosorosea* CCM 8367 and efficacy comparison with the Apopka 97 strain

Lethal concentrations (LC_50_ and LC_90_) of CCM 8367 blastospores were estimated from cumulative mortality of the last-instar-larvae of CPB at four concentrations ranging from 5×10^4^ to 5×10^7^ spores/ml of suspension. The efficacy of CCM 8367 strain was compared with commercial Apopka 97 strain applied to the larvae at concentration 5×10^7^ spores/ml of suspension. Control was treated by distilled water and Tween 80 at a 0.02% concentration. The bioassay was performed as described above.

#### The efficacy of *S*. *feltiae* against immature stages of *L*. *decemlineata*

Last-instar larvae, pre-pupae and pupae of CPB were individually placed into the Petri dishes, and then 500 IJ in distilled water were added to each dish. The control group was treated with distilled water only. Dead individuals were collected daily and cadavers were incubated for 2–3 days at the same conditions to allow nematodes to develop into adults. Cadavers were then rinsed in water to remove nematodes from the surface and then dissected in a sterile Petri dish. The number of nematodes inside each cadaver was recorded to evaluate a penetration rate.

#### The efficacy of the combined application of *I*. *fumosorosea* CCM 8367 and *S*. *feltiae* against *L*. *decemlineata* larvae

CPB last-instar larvae were immersed into suspension of CCM 8367 blastospores and individually placed into Petri dishes as described above, and 1 ml of the CCM 8367 blastospore suspension was applied topically to the larvae. Then, 500 IJ of *S*. *feltia*e in distilled water were added in the middle of each Petri dish either immediately, 24, 48 or 72 hours after fungus application. Control was treated by distilled water and Tween 80 at a 0.02% concentration. Infection symptoms and the number of dead larvae in each treatment and control were recorded daily.

#### The effect of the CCM 8367 strain on the penetration rate and the size of *S*. *feltiae* that developed inside *L*. *decemlineata* larvae

Dead CPB larvae from the combined application bioassays were incubated for 2–3 days and the *S*. *feltiae* penetration rate was evaluated as described above. Next, the adults of the first generation of nematodes that developed inside cadavers were randomly selected, collected, rinsed in water and their body size (body length and maximal body width) was measured using a Carl Zeiss (Jena, Germany) light microscope at 40×. In addition, the body size of *S*. *feltiae* developed in the last-instar CPB larvae inoculated only with nematodes was also measured.

#### Histopathology

Development and growth of *I*. *fumosorosea* CCM 8367 and *S*. *feltia*e in CPB tissue were investigated using histopathological techniques. Ten dead larvae from the experiment in which nematodes were applied simultaneously with fungus, were collected 48 and 72 hours after the treatment and put into alcoholic Bouin’s fixative solution, in a graded series of ethyl alcohol, cleared in methyl benzoate and embedded in paraffin with a high melting point. Next, 10 μm thick longitudinal sections of the larvae were cut with a Leica RM 2165 (Leica Biosystems, Nussloch, Germany) rotator microtome. Sections were then put on microscope slides and prepared according to Mallory’s procedure [42,43]. Stained sections were mounted in Canada balsam (Permount Fisher synthetic medium) and dried at room temperature [42,43]. Development of both the fungus and nematodes was investigated using an Olympus BX51 (Tokio, Japan) light microscope, and micrographs were taken with an Olympus DP50 (Tokio, Japan) digital camera attached to the microscope.

### Data presentation and statistical analysis

The obtained mortality data were corrected for mortality in the control group using the Abbott equation [44]. The Kaplan–Meier product limit estimate calculated in the LIFETEST procedure in SAS/STAT [45] was used to determine both the mean and the median time to death (LT_50_, the number of days until 50% of insects were dead) for each treatment. Wilcoxon and log-rank test statistics (PROC LIFETEST [45]) were used to test the global hypothesis that mortality (time to death) differed between treatments. Significance was set at α⩽0.05, and where multiple comparisons were performed, the Holm-Bonferroni correction [46] was applied. Data from dose-response experiment were analysed using Probit analysis (PROC PROBIT [45]) to estimate lethal concentrations (LC_50_ amd LC_90_). The penetration rate of *S*. *feltiae* was calculated using the equation P = N*100/*T*, in which *P* is a percentage of penetration, *N* is a number of nematodes counted in a cadaver, and *T* is an original number of nematodes used in the treatment. Since percentage data are not normally distributed, the effects of both the CPB developmental stage and the delay of *S*. *feltiae* application on the *S*. *feltiae* penetration rate were analyzed using a Kruskal-Wallis test [47] followed by Dunn's multiple comparison post test (PROC NPAR1WAY [45] and SAS macro [48]). Multivariate analysis of variance (MANOVA) using the Generalized Linear Models procedure (PROC GLM) in SAS [45] was used to analyze data on the body size of adult *S*. *feltiae* developed in *I*. *fumosorosea*-infected larvae. In addition, a correlation analysis of the body size data was conducted, and a model describing the relationship between the delay of nematode application and the length of *S*. *feltiae* was fitted by linear regression.

## Results

### The efficacy of *I*. *fumosorosea* CCM 8367 against immature stages of *L*. *decemlineata*

**Fig 1:**
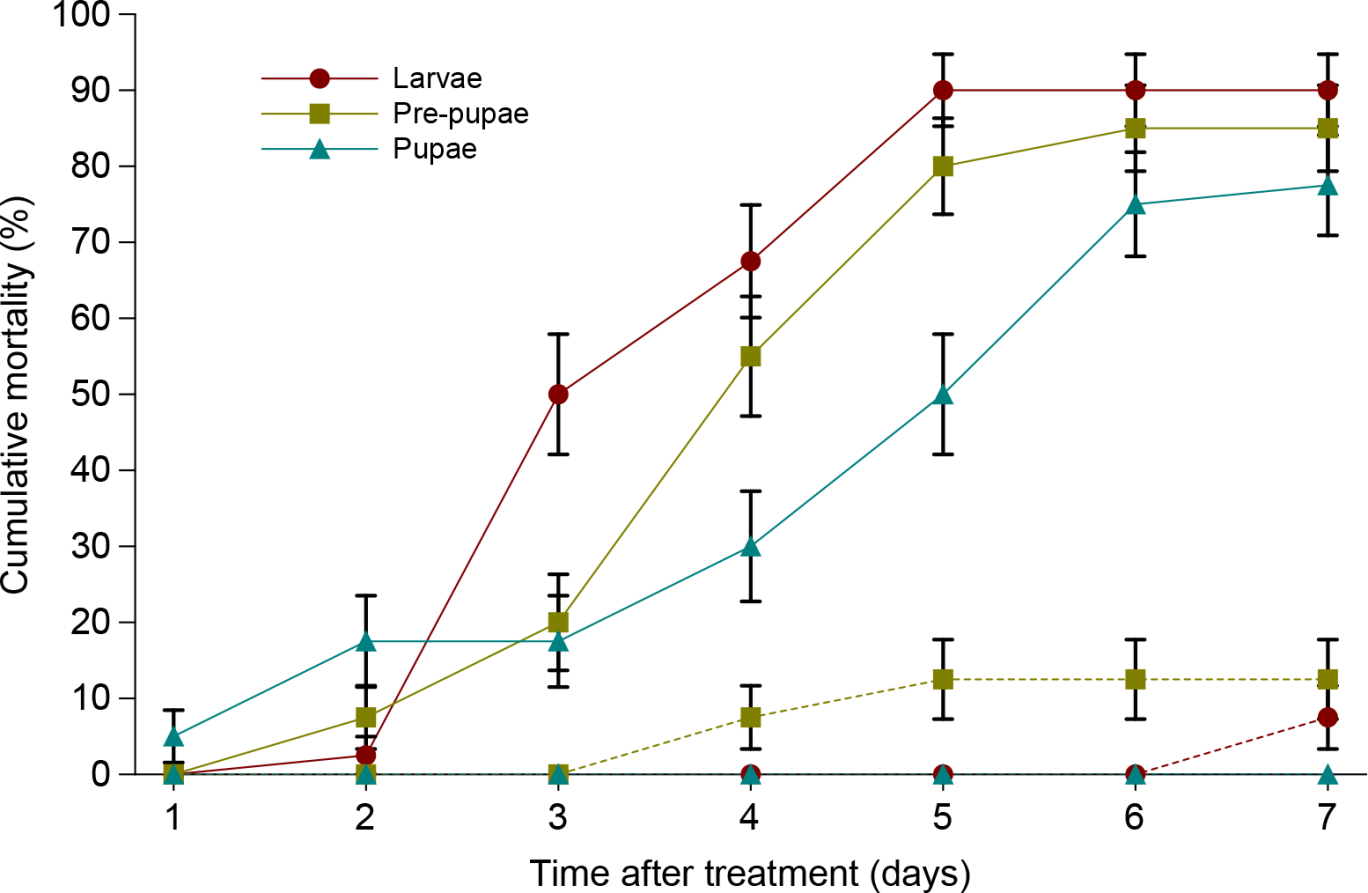
Cumulative mortality of last-instar larvae, pre-pupae and pupae of *L*. *decemlineata* treated by *I*. *fumosorosea* CCM 8367. Dashed lines indicate mortality in controls. Vertical bars indicate standard error.

The last-instar larvae of CPB showed the highest susceptibility to *I*. *fumosorosea* strain CCM 8367 followed by pre-pupae and pupae. Cumulative mortality at the end of experiment reached 90.0, 85.0 and 77.1% in last-instar larvae, pre-pupae and pupae, respectively (Fig 1). Survival analysis revealed a significant effect of developmental stage on susceptibility to the fungus (Wilcoxon test, χ^2^ = 11.07, P = 0.0039; log-rank test, χ^2^ = 9.38, P = 0.0092). Table 1 shows corrected mortality, the mean and median survival times and the associated statistical multiple comparison. All dead individuals showed symptoms of *I*. *fumosorosea* infection by mycelia growing on a cadaver usually four to five days after inoculation.

**Table 1:** Corrected mortality (%), mean survival time and LT_50_ (days) of *L*. *decemlineata* immature stages treated by suspensions of 5×10^7^ blastospores/ml of *I*. *fumosorosea* CCM 8367.

Treated Stage	Mortality^a^	Mean survival time ± SE^b^	LT_50_ (95% CI)	N^c^	Wilcoxon test^d^
Last-instar larva	89.2	3.80±0.15	3.5 (3.0–4.0)	40	a
Pre-pupa	82.9	4.38±0.19	4.0 (4.0–5.0)	40	ab
Pupa	77.5	4.80±0.27	5.0 (5.0–6.0)	40	b

^a^ Percent of dead individuals at the end of experiment corrected for mortality in control using Abbott equation [44].

^b^ The mean survival time and its standard error were underestimated because the largest observation was censored.

^c^ Total number of individuals in bioassay.

^d^ Identical lowercase letters within a column indicates no significant differences at α = 0.05 adjusted according to the Holm-Bonferroni method for multiple comparisons [46].

### Dose-response of CPB larvae to *I*. *fumosorosea* CCM 8367 and efficacy comparison with the Apopka 97 strain

**Fig 2:**
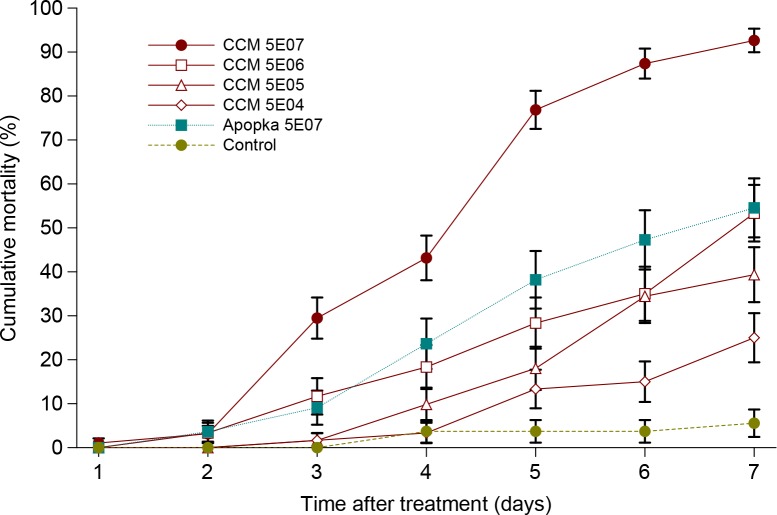
Cumulative mortality of *L*. *decemlineata* last-instar larvae treated by CCM 8367 and Apopka 97 strains of *I*. *fumosorosea*. Vertical bars indicate standard error.

**Fig 3:**
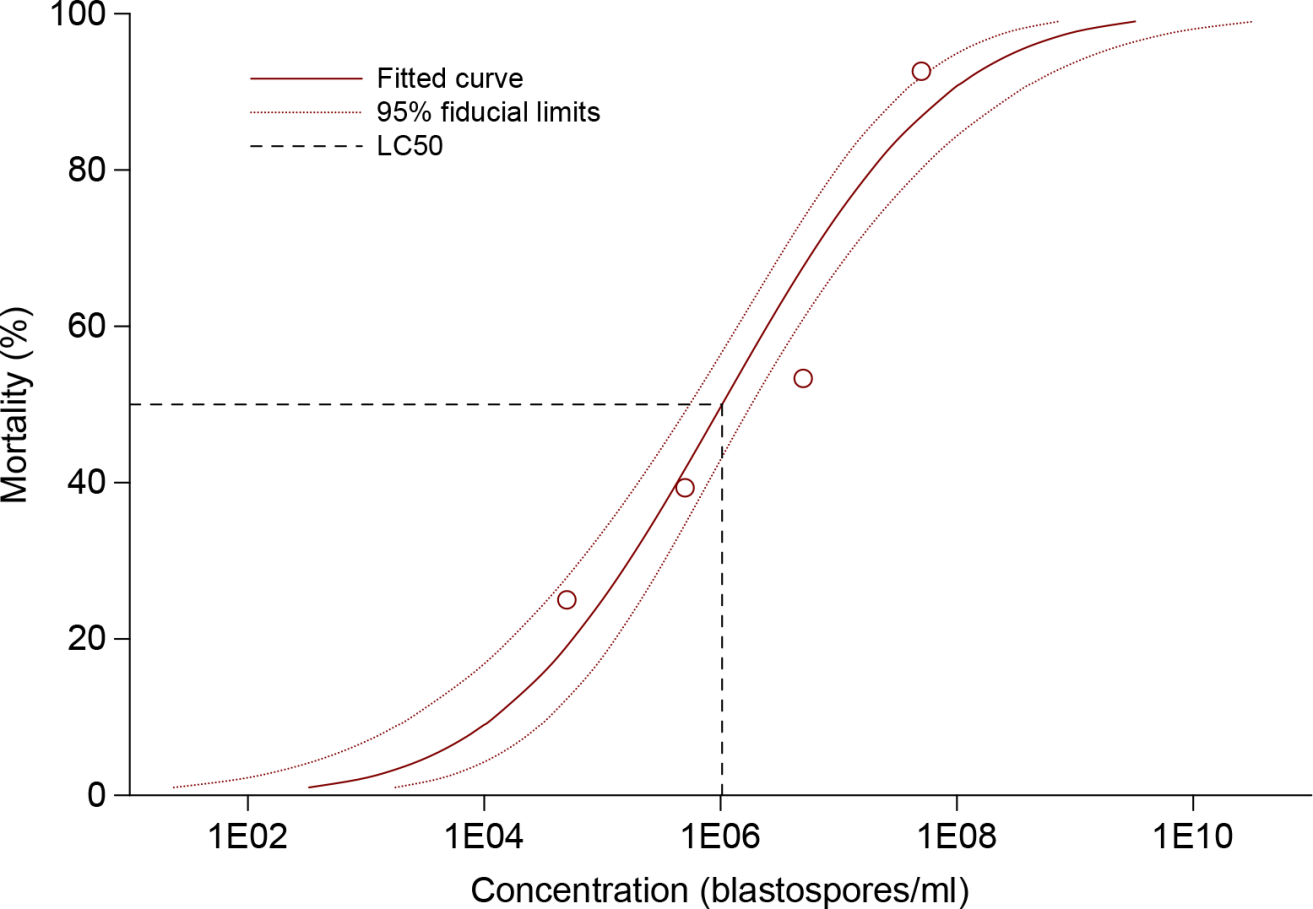
Concentration-mortality response of CPB larvae to *I*. *fumosorosea* strain CCM 8367.

Cumulative mortality of CPB larvae at the end of experiment reached 25.0, 39.4, 53.3 and 92.6 when treated by CCM 8367 strain at concentration of 5×10^4^, 5×10^5^, 5×10^6^ and 5×10^7^ blastospores/ml, respectively (Fig 2). The log-probit regression line describing relationship between concentration and mortality has a form *y* = -4.002 + 0.666*x* (Fig 3). The estimated values of LC_50_ and LC_90_ were 1.03×10^6^ and 8.67×10^7^, respectively. In Apopka 97 strain applied at concentration of 5×10^7^, cumulative mortality reached 54.5% (Fig 2) with LT_50_ 1.4 fold higher compared to CCM 8367 (Table 2). Survival analysis revealed highly significant differences in mortality rates between the two strains of *I*. *fumosorosea* (Wilcoxon test, χ^2^ = 21.85, P < 0.0001; log-rank test, χ^2^ = 27.97, P < 0.0001).

**Table 2:** Corrected mortality (%), mean survival time and LT_50_ (days) of *L*. *decemlineata* last-instar larvae treated by CCM 8367 and Apopka 97 strains of *I*. *fumosorosea*.

Strain	Dose	Mortality^a^	Mean survival time ± SE^b^	LT_50_ (95% CI)	N^c^
CCM 8367	5×10^4^	20.6	6.67±0.11	NA	60
	5×10^5^	35.8	6.36±0.14	NA	61
	5×10^6^	50.6	6.03±0.20	7.0 (7.0-NA)	60
	5×10^7^	92.2	4.59±0.15	5.0 (4.0–5.0)	95
Apopka 97	5×10^7^	51.9	5.78±0.21	7.0 (5.0-NA)	55

^a, b, c^ For explanations see Table 1.

### The efficacy of *S*. *feltiae* against immature stages of *L*. *decemlineata*

**Fig 4:**
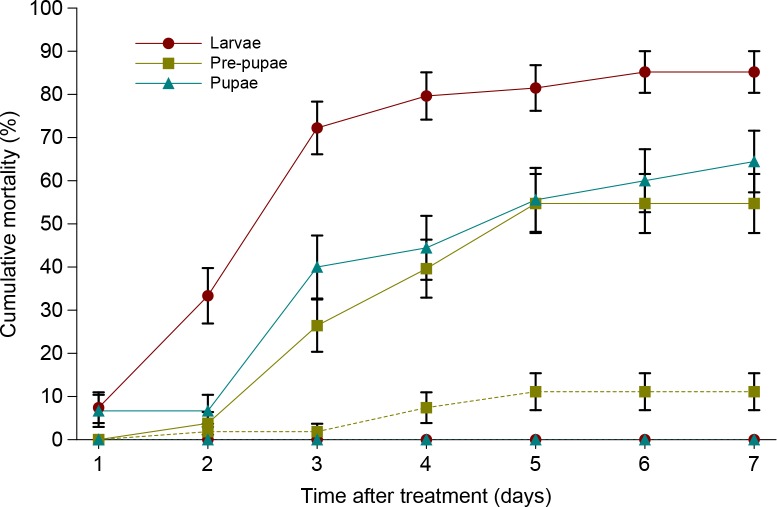
Cumulative mortality of last-instar larvae, pre-pupae and pupae of *L*. *decemlineata* treated by *S*. *feltia*e. Dashed lines indicate mortality in controls. Vertical bars indicate standard error.

*S*. *feltia*e was able to invade, develop and cause high mortality in all tested developmental stages of CPB (Fig 4). The most effective was against the last larval instar of CPB when the percentage of uncorrected mortality was 85.2%, whereas mortality of pre-pupae and pupae was 54.7.7% and 64.4%, respectively. Survival analysis revealed a significant effect of developmental stage on susceptibility to nematodes (Wilcoxon test, χ^2^ = 29.40, P < 0.0001; log-rank test, χ^2^ = 24.64, P < 0.0001) indicating differences in survival times (Table 3).

**Table 3:** Corrected mortality (%), mean survival time and LT_50_ (days) of *L*. *decemlineata* immature stages inoculated with 500 IJs of *S*. *feltiae*.

Treated Stage	Mortality^a^	Mean survival time ± SE^b^	LT_50_ (95% CI)	N^c^	Wilcoxon test^d^
Last-instar larva	85.2	3.26±0.21	3.0 (NA-NA)	54	a
Pre-pupa	49.1	4.30±0.13	5.0 (4.0-NA)	53	b
Pupa	64.4	4.87±0.31	5.0 (3.0–7.0)	45	b

^a, b, c, d^ For explanations see Table 1.

The mean number of nematodes that successfully invaded the host and developed into adults inside cadavers and the calculated penetration rates are shown in Table 4. The highest percentage of invaded IJ of *S*. *feltiae* was found in last-instar larvae. Besides normal adults, also fertilised females and first stage juveniles of the second generations were observed in some cadavers. The Kruskal-Wallis test revealed a highly significant effect of developmental stage of CPB on the penetration rate of IJ (χ^2^ = 44.70, DF = 2, P<0.0001).

**Table 4:** Mean number of *S*. *feltiae* found in cadavers of *L*. *decemlineata* immature stages inoculated with 500 IJs per dish and calculated penetration rates.

Treated stage	Number of *S*. *feltiae*	Sex ratio^a^	Penetration rate (%)	N^c^
	mean ± SE		mean ± SE^b^	
Last-instar larva	133.39 ± 7.66	54.94	26.68 ± 1.53a	46
Pre-pupa	68.39 ± 6.06	62.36	13.68 ± 1.21b	31
Pupa	68.31 ± 6.64	61.99	13.66 ± 1.33b	42

^a^ Percentage of females out of all adults.

^b^ Values followed by identical lowercase letters within a column are not significantly different at α = 0.05 (Dunn's multiple comparison test).

^c^ Number of CPB cadavers dissected.

### The efficacy of a combined application of *I*. *fumosorosea* CCM 8367 and *S*. *feltiae* against *L*. *decemlineata* larvae

**Fig 5:**
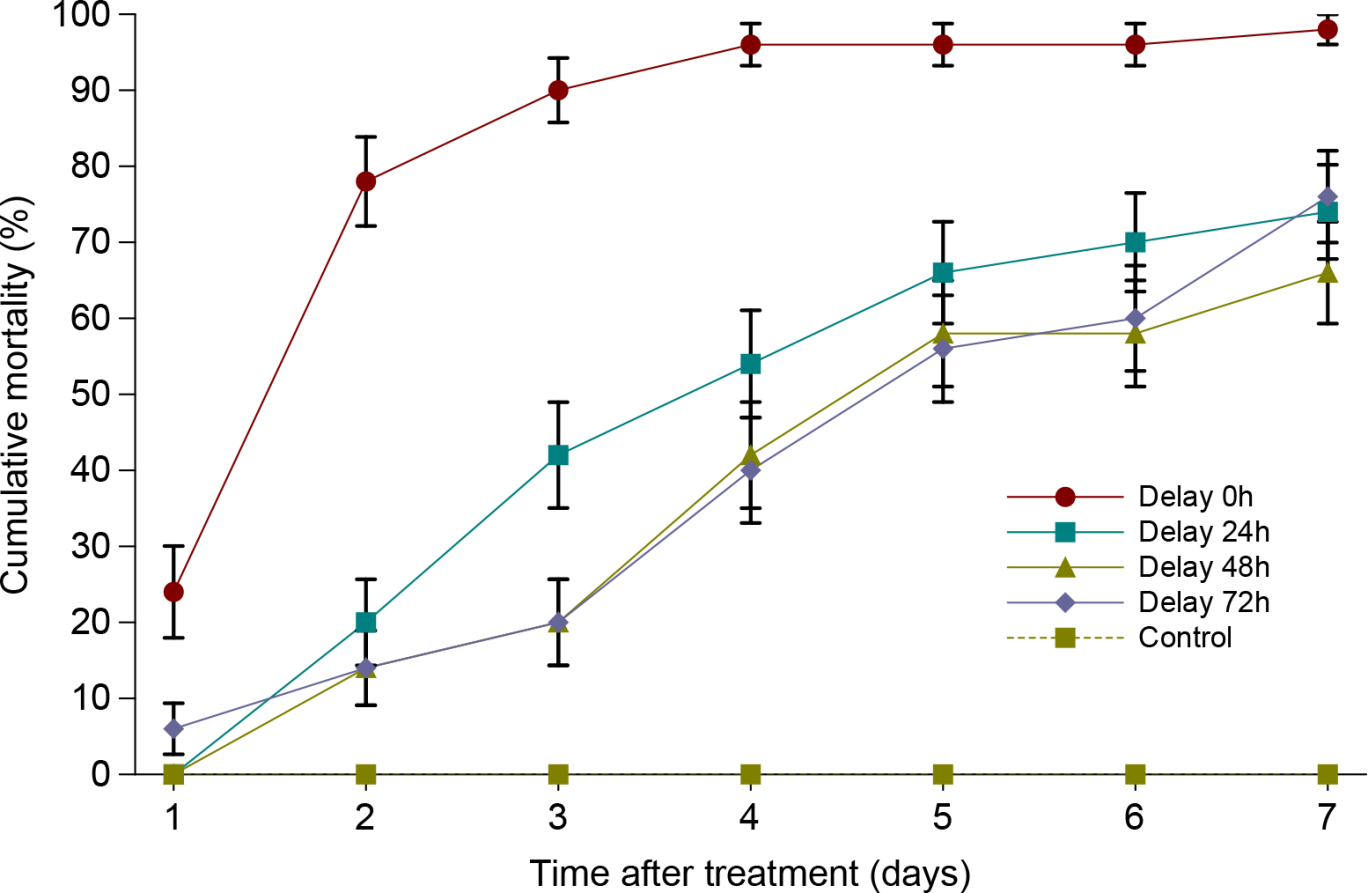
Cumulative mortality of *L*. *decemlineata* last-instar larvae treated by *I*. *fumosorosea* CCM 8367 in combination with *S*. *feltia*e. Vertical bars indicate standard error.

The results of the trials in which *S*. *feltiae* inoculation was delayed 0–72 hours after *I*. *fumosorosea* CCM 8367 application are shown in Fig 5. The percentage of mortality of the last-instar CPB larvae treated simultaneously by the nematodes and the fungus reached 78% 48 hours after the treatment and increased to 98% on the seventh day. Only symptoms of the nematode infection appeared on dead larvae in this treatment. When nematodes were applied 24 hours after the fungus, the increase of mortality was slower and total cumulative percentage of mortality reached 70% seven days after the treatment. The mortality of larvae treated by nematodes 48 hours after fungus application reached 66%. When nematodes were applied 72 hours after the fungus, larval mortality was 76% at the end of experiment. Survival analysis revealed a highly significant effect of delay between both agents’ applications on mortality of CPB larvae (Wilcoxon test, χ^2^ = 90.68, P < 0.0001; log-rank test, χ^2^ = 84.04, P < 0.0001). Multiple comparisons revealed differences between simultaneous application and delayed applications (Table 5).

**Table 5:** Corrected mortality (%), mean survival time and LT_50_ (days) of *L*. *decemlineata* last-instar larvae treated by *I*. *fumosorosea* CCM 8367 in combination with *S*. *feltia*e.

Delay of *S*. *feltiae* application (hrs)	Mortality^a^	Mean survival time ± SE^b^	LT_50_ (95% CI)	N^c^	Wilcoxon test^d^
0	98.0	2.20±0.18	2.0 (NA-NA)	50	a
24	74.0	4.48±0.28	4.0 (3.0–5.0)	50	b
48	66.0	5.08±0.26	5.0 (4.0–7.0)	50	b
72	76.0	5.04±0.28	5.0 (4.0–7.0)	50	b

^a, b, c, d^ For explanations see Table 1.

### The effect of the CCM 8367 strain on the penetration rate and the size of *S*. *feltiae* that developed inside *L*. *decemlineata* larvae

Dissection of dead CPB larvae revealed lower number of *S*. *feltiae* per cadaver (Table 6) compared to a single-agent bioassay (Table 4). This was obvious mainly when nematodes were applied 24 hours and later after fungus inoculation where IJs failed to develop to adult stages in part of the cadavers. The effect of *S*. *feltiae* application delay on the penetration rate of IJ was statistically highly significant (Kruskal-Wallis, χ^2^ = 46.00, DF = 3, P<0.0001). Similarly, the percentage of cadavers where only dead nematode adults were found increased with delay in nematode application after fungus. Sex ratio of *S*. *feltiae* in cadavers, however, was not affected (Table 6).

**Table 6:** Mean number of *S*. *feltiae* found in cadavers of *L*. *decemlineata* last-instar larvae inoculated with 500 IJs per dish in different times after the fungus application, calculated penetration rates and number of cadavers with either alive or dead nematodes.

Delay of *S*. *feltiae*	Number of *S*. *feltiae*	Sex ratio^a^	Penetration rate (%)	N^c^	N_sf_ ^d^	
application (hrs)	mean ± SE		mean ± SE^b^		Alive	Dead
0	59.60 ± 8.05	51.07	11.92 ± 1.61a	25	25	0
24	18.91 ± 4.08	46.75	3.78 ± 0.82b	23	7	1
48	7.09 ± 1.72	50.00	1.42 ± 0.34b	23	1	4
72	16.43 ± 5.08	49.46	3.29 ± 1.0b	23	2	3

^a, b, c^ For explanations see Table 4.

^d^ Number of cadavers with *S*. *feltiae* adults either alive or dead.

**Fig 6:**
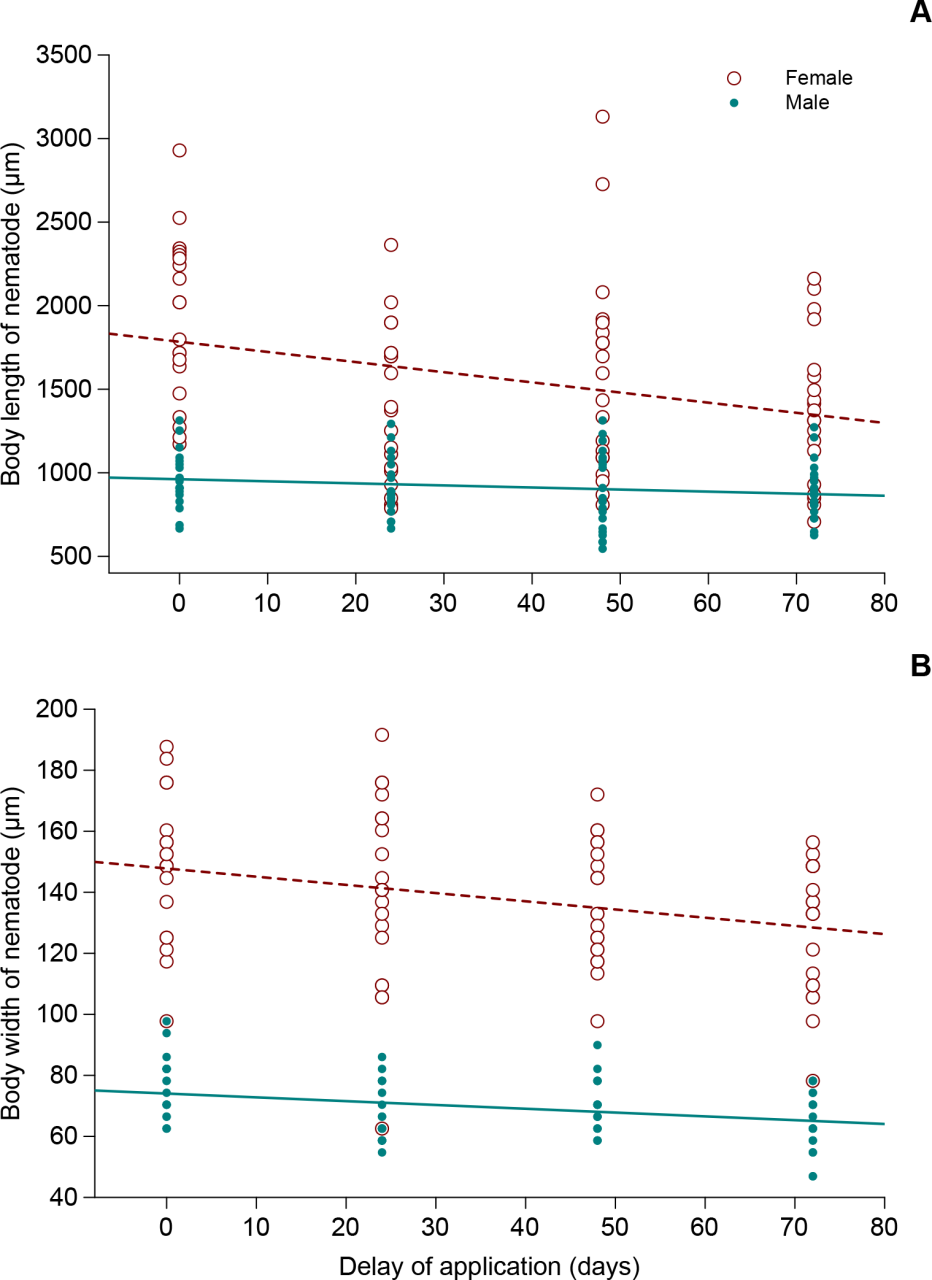
Linear regressions between the body size parameters and nematode inoculation delay. Body length (A) and body width (B) of *S*. *feltiae* adults developed inside cadavers of *L*. *decemlineata* last-instar larvae and following *I*. *fumosorosea* CCM 8367 application.

The size of first-generation *S*. *feltiae* adults recovered from CPB larvae treated simultaneously with both the nematode and *I*. *fumosorosea* was similar to the control, i.e., without fungus (Table 7). In other trials, however, nematode size decreased with the increasing time between fungus and nematode application. Results of the MANOVA indicate that there was a highly significant overall delay effect (Wilk’s λ = 0.881, F_6,308_ = 3.36, P = 0.0032) and a highly significant overall effect of sex (Wilk’s λ = 0.205, F_2,154_ = 298.76, P < 0.0001). The effect of application delay on body length and width of *S*. *feltiae* males and females inside of *I*. *fumosorosea*-treated CPB larvae is shown in Fig 6. A linear correlation analysis showed that the body length of *S*. *feltiae* females was negatively correlated with the nematode application delay (R^2^ = 0.0941; N = 80; P = 0.0056). The relationship can be described by model *y* = 1783.86–6.07*x*. A similar trend was found in the female body width (R^2^ = 0.0846; N = 80; P = 0.0089; *y* = 147.80–0.27*x*) and male body width (R^2^ = 0.1101; N = 80; P = 0.0026; *y* = 74.04–0.12*x*). No significant correlation was found in body length of males (R^2^ = 0.0302; N = 80; P = 0.1231).

**Table 7:** Mean body length and body width (μm±SE) of *S*. *feltia*e adults recovered from cadavers of *L*. *decemlineata* last-instar larvae infected by *I*. *fumosorosea* CCM 8367 and inoculated with 500 IJs per dish in different times after the fungus application.

Delay of *S*. *feltiae* application (hrs)	Females			Males		
	Length	Width	N	Length	Width	N
0	1907.89±108.13	148.19±5.16	20	977.68±39.66	76.05±2.26	20
24	1415.01±101.68	139.98±7.01	20	917.08±37.60	67.06±2.07	20
48	1566.51±136.73	136.46±4.38	20	881.73±53.87	69.99±1.92	20
72	1371.58±96.52	127.86±4.82	20	890.82±38.01	65.10±2.17	20
Control^a^	1671.55±37.31	172.04±2.89	12	1026.83±28.00	94.17±2.87	12

^a^ *S*. *feltiae* from larvae not treated by *I*. *fumosorosea*.

### Histopathology

**Fig 7:**
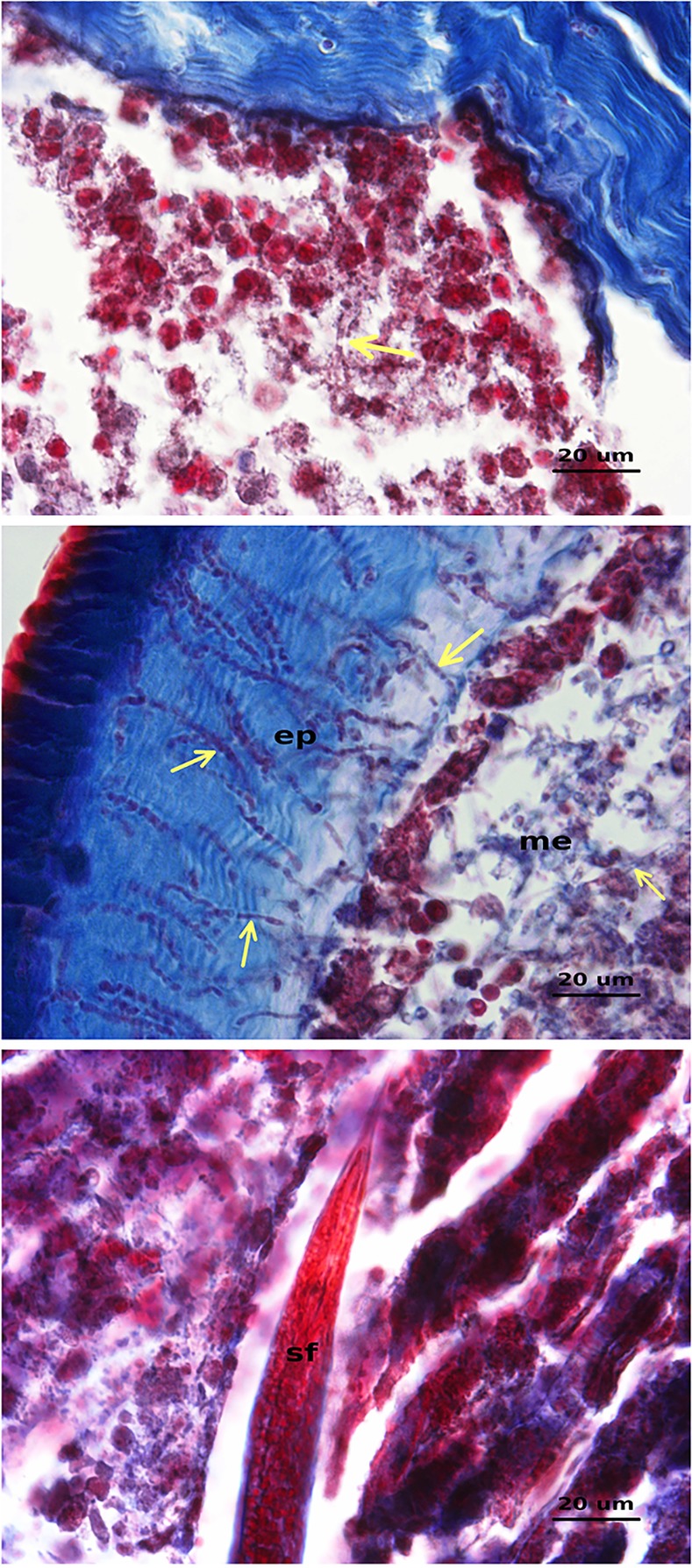
Longitudinal sections of *L*. *decemlineata* last-instar larvae infected with both *I*. *fumosorosea* and *S*. *feltia*e simultaneously. (A) Insect tissues with a few hyphal bodies of *I*. *fumosorosea* (yellow arrows) 48 hours after the treatment. (B) Hyphal bodies of *I*. *fumosorosea* (yellow arrows) penetrating epidermis (ep) and mesoderm tissues (me) 72 hours after the treatment. (C) Front part of a fertile adult female of *S*. *feltiae* (sf) full of eggs inside of tissue thoroughly invaded by hyphae of *I*. *fumosorosea* 72 hours after the treatment.

Longitudinal sections of CPB last-instar larvae showed the development of infection by *I*. *fumosorosea* CCM 8367 inside larvae 48 hours after the treatment (Fig 7A). Twenty-four hours later the fungus invaded all insect tissues (Fig 7B) whereas the development of *S*. *feltiae* was not affected by *I*. *fumosorosea* infection in CPB cadavers (Fig 7C).

## Discussion

### The efficacy of *I*. *fumosorosea* against *L*. *decemlineata*

Numerous trials have been conducted that use entomopathogenic fungi against the CPB. For example, the effect of *B*. *bassiana* on foliage consumption by fourth-instar CPB was studied by Fargues *et al*. [49]. The treated larvae consumed significantly less than control larvae, and increasing the fungus dose reduced the feeding period. The highest reduction in total food consumption per larva caused by *B*. *bassiana* was 76.2% at a dose 10^5^ conidia/cm^2^. Wraight and Ramos [50] evaluated the effects of various spray-application parameters on the efficacy of *B*. *bassiana* foliar treatments against CPB larvae during three field seasons. All treatments applied against late instars, including sprays at weekly intervals and from above the canopy, resulted in significant reductions (53–84%) of first-generation adult beetle populations. Another species of entomopathogenic fungus successfully tested against CPB was *I*. *farinosa*. In various experiments, including field trials with this species alone and in combination with other fungi performed in Poland [51,52], the Czech Republic [53] and in Austria [54], high efficacy of fungus treatment was reported.

The present research studied the efficacy of *I*. *fumosorosea* against the immature stages of CPB. The species has a worldwide distribution, and its natural occurrence in soil samples was reported from many countries. For example, in the Czech Republic, 16 strains of *I*. *fumosorosea* were isolated from various soils by Landa *et al*. [55]. In another soil-sample survey [56], *I*. *fumosorosea* was the dominant species (77.6%) whereas *I*. *farinosa* occurred rarely (1.7%). The species has a relatively wide host range across several insect orders, including Acari [12]. It was also reported to infect *L*. *decemlineata* [57].

The strain CCM 8367 used in this study was isolated from *C*. *ohridella*, an invasive pest of horse chestnut in Europe [58]. The first record of *I*. *fumosorosea* infecting pupae of this host was reported by Zemek *et al*. [59]. Because this strain showed high virulence against *C*. *ohridella* and other pests [60–62], it was considered to have an application potential and therefore patented [39].

Our study demonstrates a high efficacy of the CCM 8367 strain of *I*. *fumosorosea* against all three immature stages of CPB tested. The most virulent was against the CPB last-instar larvae when it caused nearly 93% mortality 7 days after the treatment by suspension of blastospores with a concentration of 5×10^7^ spores/ml. Strain Apopka 97 at the same concentration caused significantly lower mortality of CPB larvae. This strain originates from a mealybug *Phenacoccus sp*. in Apopka, Florida, USA [63], and several studies have demonstrated its high efficacy against whiteflies [64,65], psyllids [66,67] and thrips [68]. Presently, it is readily available as a commercial product in the USA (PFR 97^TM^, Certis Columbia, MD) and Europe (PreFeRal® WG, Biobest, Belgium).

### The efficacy of *S*. *feltiae* against *L*. *decemlineata*

The application of EPNs in biological control was traditionally used to control soil pests [69]. Research from the last two decades also indicates their potential against foliar pests, but only under special conditions [70,71]. The results of the present study show that *S*. *feltiae* caused medium to high mortality in immature stages of CPB. The highest efficacy (85.2%) was found when nematodes were applied to last-instar larvae. This is consistent with the well-known fact that EPNs are most effective in controlling younger developmental stages because entering the host is much easier [72,73]. *Steinernema carpocapsae* (Weiser), *S*. *feltiae* and *Heterorhabditis bacteriophora* Poinar strains applied at a dose of 164.6 nematodes/cm^2^ of soil were able to kill 100% of CPB pre-pupae under laboratory conditions [74]. Other laboratory experiments showed that adults of *L*. *decemlineata* are also sensitive to EPNs [32,73]. The efficacy of two strains of *S*. *feltiae* against CPB was tested in a field experiment by Laznik *et al*. [75]. Both strains significantly decreased the number of larvae, whereas no effect on CPB eggs and adults was observed.

The percentage of nematodes invading CPB immature stages (penetration rate) was low, ranging from 13.7% to 26.7%. These results are similar to the findings of Epsky and Capinera [76] who observed that 10–50% of applied *S*. *carpocapsae* successfully infected the host. The percentage of invading nematodes of *S*. *feltiae* and *H*. *bacteriophora* to CPB under laboratory and greenhouse conditions ranged between 10–50% [77]. The study performed by Armer *et al*. [78] showed that although *Heterorhabditis marelatus* Liu and Berry is capable of successfully attacking and killing CPB, the nematode is incapable of completing its life cycle in the beetle. This phenomenon was later attributed to stress on the nematode symbiont *Photorhabdus temperata* Fischer-Le Saux, Viallard, Brunel, Normand and Boemare and potential interference from the enteric bacteria of the beetle [79]. Similarly, Campos-Herrera and Gutierrez [80] reported that neither *S*. *feltiae* was able to reproduce in CPB. In our experiments, we did not study the ability of *S*. *feltiae* to reproduce in CPB, however during the dissections, we did not observe any negative effect on the first generation adults.

### The efficacy of the combined application of *I*. *fumosorosea* CCM 8367 and *S*. *feltiae* against *L*. *decemlineata* larvae

The combination of *I*. *fumosorosea* CCM 8367 with nematodes increased the application efficiency compared to single biocontrol agent application. The best results were obtained when *S*. *feltiae* was applied simultaneously with *I*. *fumosorosea*. A synergistic effect was reported, e.g., when entomopathogenic nematodes were applied together with *Paenibacillus popilliae* [81,82], or with *Bacillus thuringiensis* Berliner subspecies *japonensis* against *Cyclocephala* spp. [83,84]. Another study [85] demonstrated that the application of *B*. *bassiana* with *H*. *bacteriophora* resulted in higher total mortality of *Spodoptera exigua* (Hübner) (Lepidoptera: Noctuidae) in soil than when either nematodes or fungi were separately applied. However, when the same fungus species was combined with *S*. *carpocapsae*, insect mortality was not significantly different compared with *S*. *carpocapsae* alone [85]. By contrast, Shapiro-Ilan *et al*. [86] found that when pairs of nematode and fungal pathogens attacked the larvae of the weevil *Curculio caryae*, most pairings were less effective than a single highly effective entomopathogenic species. This antagonism may have resulted from negative interactions between microbes or their toxins before or during the infection process.

Our results demonstrate a normal development of nematodes when they were applied simultaneously with the fungus. Similar conclusions were reported in other studies showing that when *B*. *bassiana* and *S*. *carpocapsae* or *H*. *bacteriophora* were applied simultaneously to a host, the nematodes developed normally and produced progeny [87,88]. When, however, *S*. *feltiae* was applied more than 24 hours after *I*. *fumosorosea* CCM 8367 treatment, its development was negatively affected. Only a part of the nematodes developed to adults, and in some cadavers, only dead adults were observed. Furthermore, adult body size expressed as body length and width of nematodes developing inside the cadavers in these treatments was lower in comparison to control. This could be because of the anti-bacterial activity of some metabolites/toxins produced by *I*. *fumosorosea* that negatively affect both the developing nematodes and their symbiotic bacteria, *Xenorhabdus bovienii*. A similar negative effect was observed in the interactions between the fungi *M*. *anisopliae* and *S*. *glaseri* [89] and *H*. *bacteriophora* [90] and between *B*. *bassiana* and *S*. *ichnusae* [91]; however, this was not explored in our study.

### Conclusions

We found that (1) both *I*. *fumosorosea* CCM 8367 and *S*. *feltiae* Ustinov showed high virulence against *L*. *decemlineata*, (2) the most sensitive stage of CPB is the last-instar larva, (3) simultaneous application of both biocontrol agents increases their efficacy compared to single species application and (4) later application of *S*. *feltiae* has a negative effect on both the penetration rate and the development of nematodes inside a CPB host. Further research, including soil experiments in greenhouses and in the field, is necessary before these strains can be recommended for application as biopesticides.

## Supporting Information

S1 Dataset Fig 1, Table 1 original data. (XLSX)

S2 Dataset Fig 2, Fig 3, Table 2 original data. (XLSX)

S3 Dataset Fig 4, Table 3 original data. (XLSX)

S4 Dataset Table 4 original data. (XLSX)

S5 Dataset Fig 5, Table 5 original data. (XLSX)

S6 Dataset Table 6 original data. (XLSX)

S7 Dataset Fig 6, Table 7 original data. (XLSX)
